# Prolonged fever and exaggerated hypercoagulopathy in malaria vivax relapse and COVID-19 co-infection: a case report

**DOI:** 10.1186/s12936-022-04215-5

**Published:** 2022-06-23

**Authors:** Tri Pudy Asmarawati, Okla Sekar Martani, Bramantono Bramantono, Muhammad Vitanata Arfijanto

**Affiliations:** 1 Tropical and Infectious Diseases Division, Department of Internal Medicine, Faculty of Medicine, Universitas Airlangga, Surabaya, East Java 60115 Indonesia; 2 Universitas Airlangga Hospital, Surabaya, East Java 60115 Indonesia; 3 Dr. Soetomo General Teaching Hospital, Surabaya, East Java 60286 Indonesia; 4 Faculty of Medicine, Universitas Airlangga, Surabaya, East Java 60115 Indonesia

**Keywords:** COVID-19, malaria, *Plasmodium vivax*, Infectious diseases

## Abstract

**Background:**

Coronavirus disease 2019 (COVID-19) often causes atypical clinical manifestations similar to other infectious diseases. In malaria-endemic areas, the pandemic situation will very likely result in co-infection of COVID-19 and malaria, although reports to date are still few. Meanwhile, this disease will be challenging to diagnose in areas with low malaria prevalence because the symptoms closely resemble COVID-19.

**Case presentation:**

A 23-year-old male patient presented to the hospital with fever, anosmia, headache, and nausea 1 week before. He was diagnosed with COVID-19 and treated for approximately 10 days, then discharged to continue self-quarantine at home. 2 weeks later, he returned to the hospital with a fever raised intermittently every 2 days and marked by a chilling-fever-sweating cycle. A laboratory test for malaria and a nasopharyngeal swab for SARS CoV-2 PCR were conducted, confirming both diagnoses. The laboratory examination showed markedly elevated D-dimer. He was treated with dihydroartemisinin-piperaquine (DHP) 4 tablets per day for 3 days and primaquine 2 tablets per day for 14 days according to Indonesian National Anti-malarial Treatment Guidelines. After 6 days of treatment, the patient had no complaints, and the results of laboratory tests had improved. This report describes the key points in considering the differential diagnosis and prompt treatment of malaria infection during the pandemic of COVID-19 in an endemic country to prevent the worse clinical outcomes. COVID-19 and malaria may also cause a hypercoagulable state, so a co-infection of those diseases may impact the prognosis of the disease.

**Conclusion:**

This case report shows that considering the possibility of a co-infection in a COVID-19 patient who presents with fever can prevent delayed treatment that can worsen the disease outcome. Paying more attention to a history of travel to malaria-endemic areas, a history of previous malaria infection, and exploring anamnesis regarding the fever patterns in patients are important points in making a differential diagnosis of malaria infection during the COVID-19 pandemic.

## Background

Severe acute respiratory syndrome coronavirus 2 (SARS-CoV-2) is a new strain of coronavirus that started to emerge in December 2019 in Wuhan, China, and spread rapidly throughout the world, causing a coronavirus disease (COVID-19) pandemic [1]. The Indonesian government reported that the number of confirmed cases of COVID-19 on August 1, 2021, was 3,409,658, with a total number of 94,119 deaths or a case fatality rate of 2.76% [2]. The SARS-CoV-2 infection causes various clinical manifestations ranging from asymptomatic to a broad spectrum of symptoms, such as mild symptoms of upper respiratory tract disorders and life-threatening sepsis. Recent studies have also reported that COVID-19 patients frequently develop hypercoagulopathy and a high prevalence of thromboembolic events [3]. The most common feature in hospitalized COVID-19 patients is fever (70–90%), impaired sense of smell and taste (64–80%), dry cough (60–86%), shortness of breath (53–80%), fatigue (38%), nausea/vomiting or diarrhoea (15–39%), and myalgia (15–44%) [4]. Coagulopathy with abnormally elevated D-dimer levels has been reported in 260 of 560 cases of COVID-19 (46.4%), with the prevalence of 43% in non-severe patients compared with 60% in critically ill ICU patients.

Malaria is an infectious disease caused by protozoa of the genus *Plasmodium*, which is transmitted through the bite of an *Anopheles* mosquito. Malaria infections can cause severe clinical manifestations and death if not promptly diagnosed and treated. There are five species of *Plasmodium* that cause malaria in humans, with the most deaths caused by *Plasmodium falciparum. Plasmodium vivax* predominates as a cause of morbidity, while *Plasmodium ovale* and *Plasmodium malariae* rarely cause severe malaria. *Plasmodium knowlesi* primarily infects macaques in Southeast Asia, but humans living around these animals can become infected [5].

Malaria remains a serious global health problem, especially in endemic countries. In 2019, an estimated 229 million malaria cases occurred worldwide, with 3% of all cases caused by *Plasmodium vivax*, which is the predominant parasite responsible for 51.7% of malaria cases in Southeast Asia, including Indonesia [6]. Patients with malaria will usually experience paroxysmal fever, fatigue, malaise, and myalgia [7]. A co-infection between COVID-19 and malaria is rarely reported, and the mechanism is unclear. It is suggested that a co-infection leads to excess proinflammatory responses and pro-coagulant states that might result in more severe manifestations and poor prognosis. Heightened clinical suspicion is needed to diagnose other infections during the COVID-19 pandemic, especially for patients with a travelling history of malaria-endemic areas. In this case report, we found a confirmed COVID-19 patient diagnosed with malaria.

### Case presentation

**Fig. 1:**
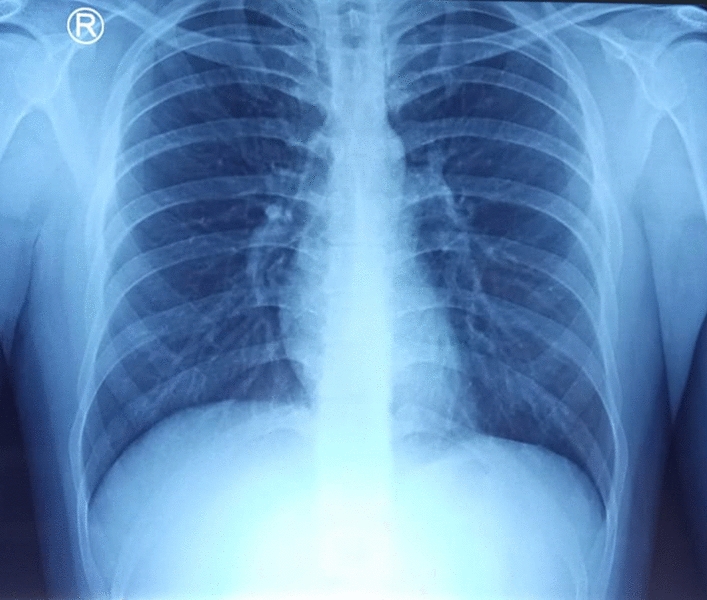
Chest x-ray on first admission

A 23-year-old male patient came to the emergency department at Universitas Airlangga Hospital presenting fever, anosmia, headache, and nausea 5 days before. He was diagnosed with COVID-19, confirmed by the RT-PCR amplification of SARS-CoV-2 virus nucleic acid on a nasopharyngeal swab. The patient had abnormal laboratory findings, such as lymphopenia, neutrophilia, thrombocytopenia, and high CRP, with a normal chest X-ray (Fig. 1). The patient was treated with Favipiravir at 1600 mg per 12 h on day 1, then 600 mg per 12 h on day 2-day 5, intravenous drip of paracetamol 1000 mg every 8 h, oral vitamin C 500 mg per 8 h, and oral vitamin D 1000 IU per day according to Indonesian national COVID-19 treatment guidelines. After 10 days of treatment, the patient was still PCR positive despite no complaints. He was then discharged to continue self-quarantine at home.

He returned to the hospital two weeks later with general weakness and fever. The fever did not resolve during his self-quarantine, was raised intermittently every 2 days, and was marked by a chilling-fever-sweating cycle. The fever temperature measured was around 39.7°–40 °C. The fever improved when the patient took paracetamol, but the fever returned 6 h later. The fever was accompanied by malaise and muscle pain. The next day the patient was fever-free and able to carry out his daily activities. The physical examination revealed a body temperature of 38 °C, a blood pressure of 112/72 mmHg, a heart rate of 93 bpm, a respiratory rate of 20 breaths/minute, and oxygen saturation of 98% under ambient air. The patient weighs 65 kg with a height of 170 cm (BMI 22.5 kg/m^2^). Other physical examinations were normal.

**Fig. 2:**
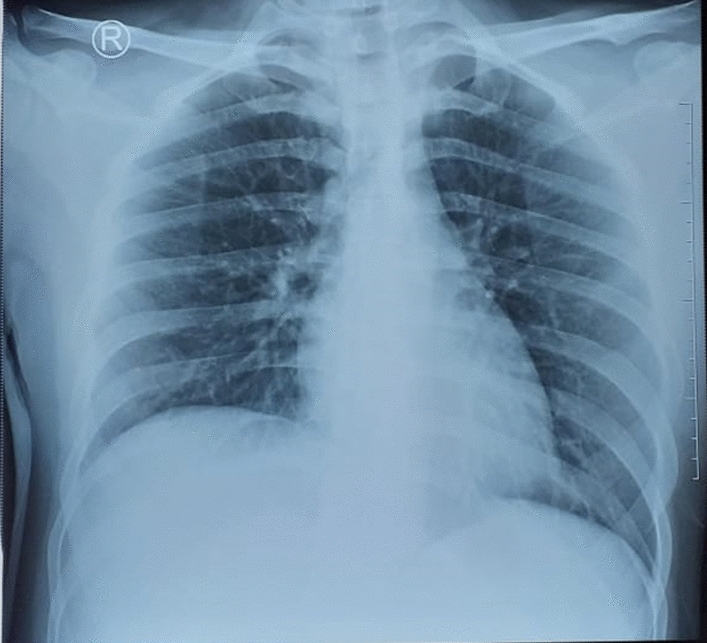
Chest x-ray on second admission

The laboratory results reflected a white blood cell count of 8750/µL with 76.3% neutrophils, 13.5% lymphocytes, and 0.6% eosinophils. Haemoglobin level and platelet counts were 12.2 g/dL and 231,000/µL, respectively. CRP and D-dimer levels were 41.51 mg/L and 9.52 mg/L, respectively. Serum electrolytes and renal function tests were normal, with urea and creatinine serum 6 mg/dL and 0.4 mg/dL, respectively. The liver enzymes test was normal, with ALT being 14 IU/L and AST being 25 IU/L. Electrocardiography showed normal sinus rhythm and axis. The chest X-ray was unremarkable (Fig. 2). The RT-PCR amplification of the SARS-CoV-2 virus nucleic acid test from the nasopharyngeal swab was still positive.

**Fig. 3:**
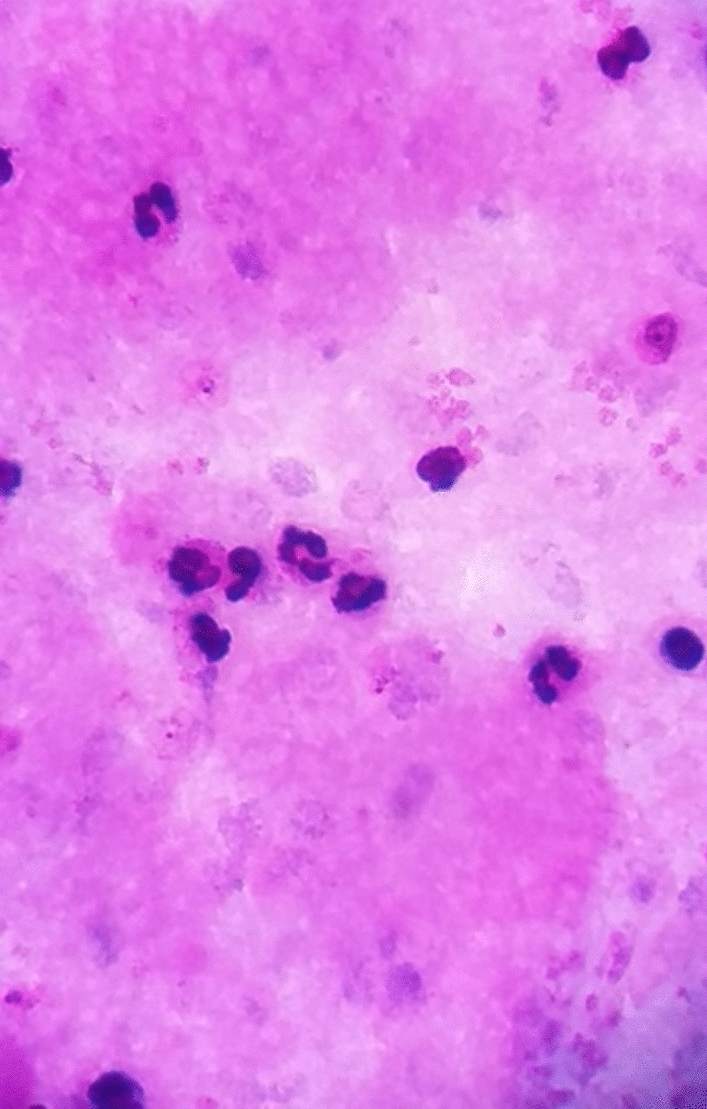
Thick blood smear

**Fig. 4:**
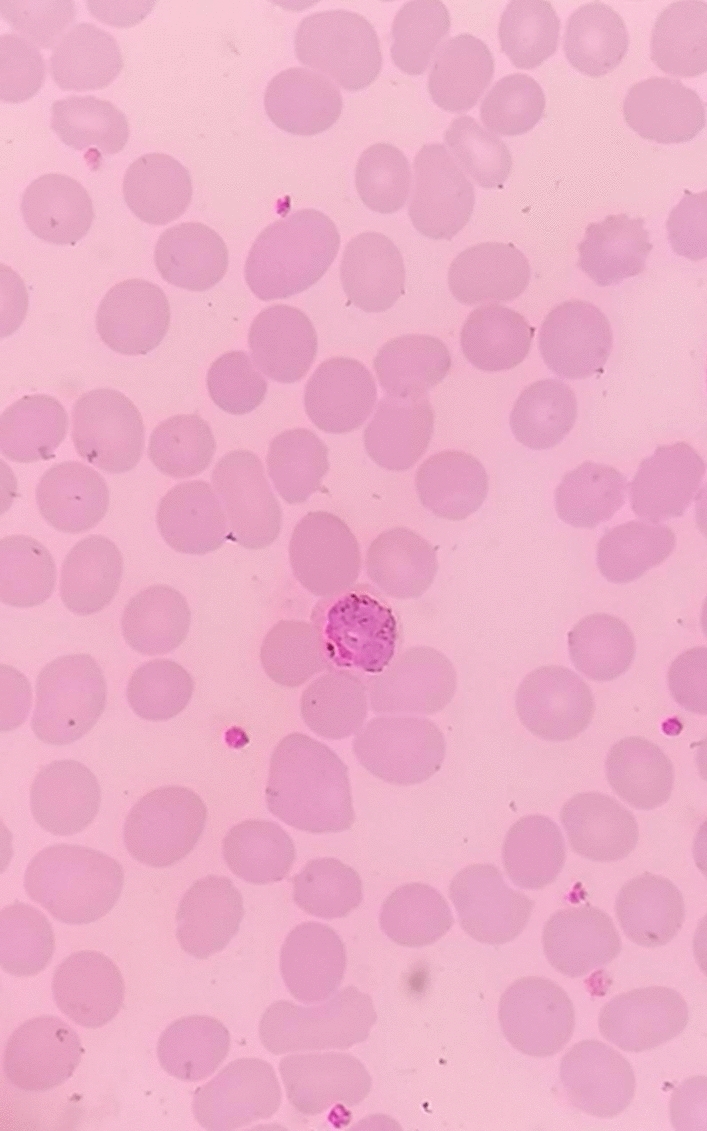
Gametocyte in thin blood smear

**Fig. 5:**
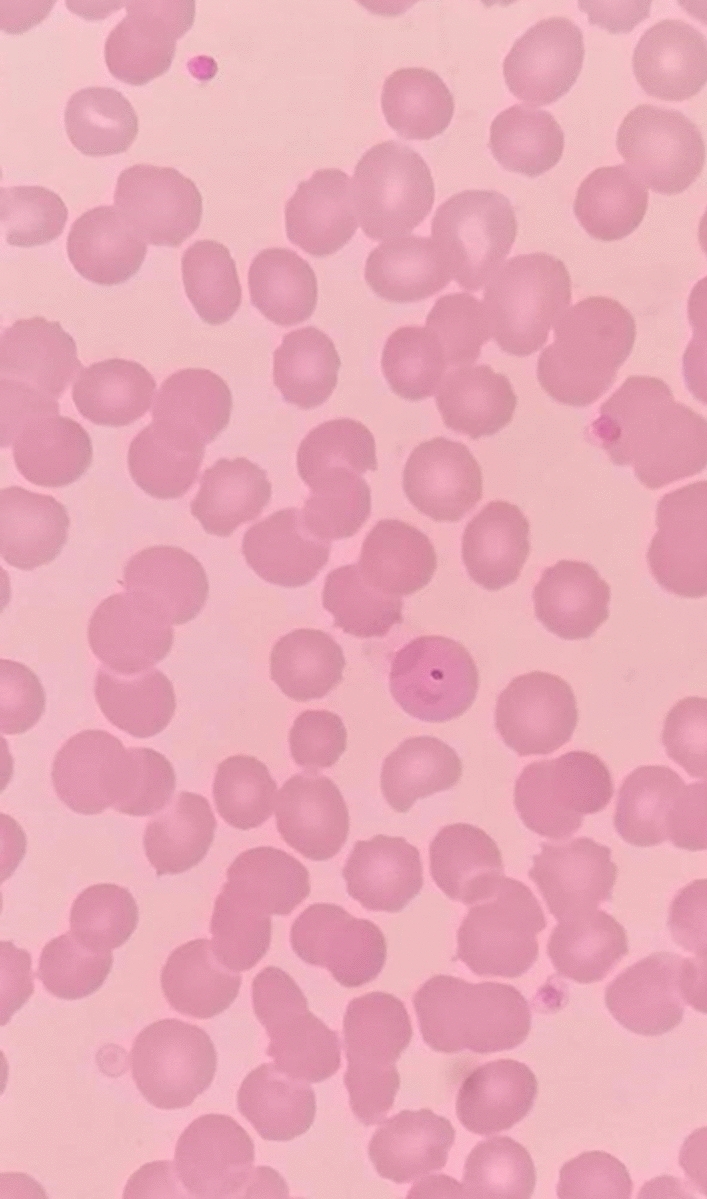
Ring stage in thin blood smear

**Fig. 6:**
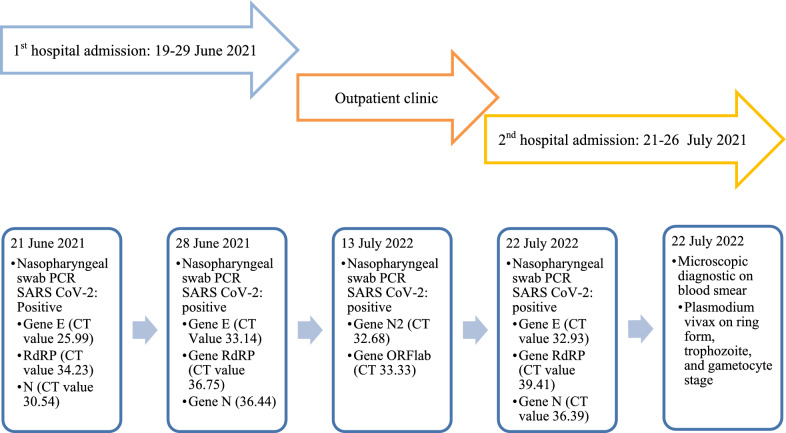
COVID-19 and Malaria relapse disease course

From the previous history, he said that he traveled to Timika District, Papua, for office work for the last 2 years. In May 2021, the patient was infected with *P vivax*, with initial symptoms including fever, dizziness, nausea, and muscle pain. The patient was treated until declared fully cured. A laboratory test for malaria was performed. The rapid malaria test was positive for PAN antigen, and microscopic diagnosis on blood smear revealed *Plasmodium vivax* on ring form, trophozoite, and gametocyte stage (Figs. 3, 4, 5). Based on anamnesis, physical examination, and laboratory results, the patient was diagnosed with confirmed COVID-19 with hypercoagulopathy and malaria vivax relapse (Fig. 6). According to Indonesian National Guidelines for Antimalarial Treatment [8], he was treated with dihydroartemisinin-piperaquine (DHP) 4 tablets per day for 3 days and primaquine 2 tablets per day for 14 days. The G6PD status was not tested. The patient was given an intravenous drip of paracetamol 1000 mg every 8 h and a subcutaneous injection of heparin 5000 IU every 12 h during treatment. After six days of treatment, the patient had no complaints, and the results of laboratory tests had improved. The patient was discharged from the hospital, continued self-isolation at home, and followed up with the internal medicine outpatient clinic two weeks later. Furthermore, the patient was called later and reported feeling healthy with no complaints.

## Discussion

Indonesia is one of the tropical countries in the world still facing a high risk of malaria. Approximately 80% of cities or districts in Indonesia are endemic to malaria, one of which is Papua. Based on the data from Annual Parasite Incidence (API) in 2019, the number of positive cases of malaria in Papua is 64.03% per 1000 population [9]. *Plasmodium vivax* malaria still causes significant morbidity in endemic areas. Reactivation of the dormant stage of hypnozoite can cause *P. vivax* relapse [10]. A single inoculation by a female *Anopheles* mosquito can be followed by multiple relapses [11].

*Plasmodium vivax* is capable of undergoing early and frequent relapses. In addition to blood-stage parasite replication, *P. vivax* has a dormant stage of hypnozoites that can persist in the liver and reactivate to cause relapses weeks or even years later [12]. This reactivation may be triggered by the host’s inflammatory response to systemic illnesses or parasitic and bacterial infections, but not viral infections [13]. The estimated ratio of *P. vivax* relapses to total infections is 76–90% in the Papua New Guinea cohort study and 79% in the Thailand cohort study [12]. Based on the study conducted by Chu et al. *P. vivax* parasitaemia recurrences, mainly relapses, occurred in 377 from 644 (59%) patients treated with artesunate, chloroquine, or chloroquine-primaquine. A history of the previous malaria was more common in patients with recurrences (61%) than without recurrence (39%). About 90% of all *P. vivax* recurrences occurred by week 16 following treatment. Primaquine, as the radical cure with an estimated efficacy of 92%, would reduce the risk of relapses and lessen the significant burden of morbidity caused by *P. vivax* [14].

The pathophysiology behind the co-infection of COVID-19 with *P. vivax* malaria remains unclear. It is unknown whether SARS-CoV-2 infection reduces the immunity that leads to malaria reactivation or if complications from malaria increase the susceptibility to get COVID-19 [15]. Based on the disease course, this patient was suspected of having reactivation of *P. vivax* infection due to COVID-19 infection. The mechanism that caused the reactivation is still unclear and may be attributed to the cytokine response to COVID-19. There is still a possibility of natural reactivation or reinfection not related to COVID-19 infection that cannot be completely ruled out [16]. The excessive proinflammatory cytokine response generated in co-infection of COVID-19 and Malaria can worsen the prognosis [17].

The patient was also suspected of having prolonged viral shedding of SARS-CoV-2; the PCR was still positive on day 38 after symptom onset. This phenomenon may be attributable to several risk factors, including male sex, disease severity, delayed hospital admission, underlying comorbidities, and a higher pro-inflammatory cytokine level [18–20]. In a large Italian cohort study, Mondi et al. stated that pro-inflammatory marker changes, especially lymphopenia, and elevated D-dimer, were strongly associated with prolonged viral shedding [19]. A higher neutrophil count and CRP were also mentioned in a report by Gao et al. as independent risk factors of prolonged viral shedding found in COVID-19 patients with bacterial co-infection [20]. Rather than bacterial infection, the abnormal proinflammatory cytokine and infection markers related to prolonged SARS-CoV-2 RNA shedding in this case were exaggerated by malaria vivax relapse.

Early symptoms of SARS-CoV-2 infection, such as fever, fatigue, and myalgia, similar to those of malaria, can lead to delayed diagnosis, especially in malaria-endemic areas [16]. In contrast to severe malaria, which is often caused by *P. falciparum*, where neurological symptoms are predominant and more severe symptoms such as loss of consciousness, signs of focal neurological abnormalities, and severe anaemia can further narrow the diagnosis of malaria. The results of blood tests can also be confusing. Therefore, a diagnostic test for both malaria and COVID-19 is crucial. The principal diagnosis of malaria to date is the microscopic examination to detect the presence of *Plasmodium* at all stages. However, the sensitivity and specificity of this examination are highly dependent on the examiner’s subjectivity. If a microscopy test is not available, malaria rapid diagnostic test (RDT) can be an alternative to confirm the diagnosis. Another method using a PCR is more sensitive than microscopy, but the results take too long, so it is rarely used [21]. *Plasmodium* spp and COVID-19 have incubation periods that are not much different. For COVID-19, the incubation period reaches 14 days from exposure with a median value of 4–5 days, while for malaria, the incubation period varies from 7 to 30 days [21]. Hence, it is essential to gain a thorough anamnesis in differentiating these two diagnoses.

Hypercoagulopathy is common in COVID-19 patients, especially in those with severe disease [22]. SARS-CoV-2 induces tissue factor expression, a primary initiator of the coagulation cascade, by cytokines produced by inflammatory cells. Moreover, SARS-CoV-2 causes endothelial dysfunction through an angiotensin-converting enzyme-2 (ACE-2) receptor expressed on the surface of vascular endothelial cells and induces neutrophil extracellular traps (NETs) release, which activates the coagulation pathways and platelet [23]. Abnormal coagulation parameters such as elevated D-dimer and fibrin degradation product levels and prolonged prothrombin time are related to a poor outcome. The most common manifestations of COVID-19 hypercoagulopathy are venous thromboembolism and arterial thrombotic complications, including pulmonary embolism and stroke [3]. COVID-19 patients are at risk for developing disseminated intravascular coagulation (DIC), pulmonary haemorrhage, and thrombosis. Thrombocytopenia associated with a higher risk of severe COVID-19 is suspected to be caused by platelet consumption in the lungs and infected haematopoietic stem cells and megakaryocytes [24].

Malaria is also strongly associated with a hypercoagulopathy condition through activation of the coagulation cascade triggered by proinflammatory cytokines [e.g., tumour necrosis factor (TNF) and interleukin (IL)-6] [25]. The most common coagulopathy condition is microthrombotic complications, besides thrombosis of large vessels, including cerebral venous thrombosis and pulmonary embolism. Thrombocytopenia is a common finding (60–80%) that may be due to impaired coagulation, splenomegaly, bone marrow disorders, antibody-mediated platelet destruction, oxidative stress, and platelet aggregation. DIC and bleeding are related to high mortality, occurring only in severe malaria. Tissue factors released from damaged vascular endothelial cells and the lysis of activated platelets contribute to the development of a pro-coagulant state similar to the underlying mechanism in COVID-19 [26]. Therefore, *Plasmodium* spp. and SARS-CoV-2 co-infection could lead to severe coagulopathy and worse outcomes than with either infection alone. In this case, the patient had a high risk of mortality due to hypercoagulopathy, characterized by markedly increased D-dimer levels which was also aggravated by malaria vivax relapse. Regarding this condition, thromboprophylaxis has a crucial role in reducing the risk of thrombotic events.

There was also some similar report regarding malaria and COVID-19 co-infection, as shown in Table 1. The first case report was described by Sardar et al. [17] in Qatar as a possible *P. vivax* reactivation secondary to COVID-19, similar to the case reports presented by Kishore et al. [16] in India and Shahid et al. [11] in Qatar. Ray et al. reported a concomitant infection between malaria vivax and COVID-19 in India without suggesting the possibility of *P. vivax* re-activation [27]. However, this report is the first case of malaria vivax and COVID-19 co-infection in Indonesia that discussed the possibility of *P. vivax* relapse related to COVID-19 with symptoms of prolonged fever and exaggerated hypercoagulopathy.

**Table 1:** A Literature review of COVID-19 and *Plasmodium vivax* Co-infection

No	Author	Country	Year of Publication	Age	Clinical symptoms	History of *P. vivax* infection
1	Shahid et al. [11]	Qatar	2021	55	Dry cough, high-grade fever, chills, rigors, profuse sweating, lethargy	Documented (1 year before)
2	Ray et al. [27]	India	2020	67	Fever, shortness of breath	Not documented
3	Kishore et al. [16]	India	2020	10	High-grade fever, chills, rigors, headache, cold, cough, abdominal pain	Documented (6 months before)
4	Sardar et al. [17]	Qatar	2020	34	Fever, myalgia, vomiting, right upper quadrant abdominal pain	Not documented (but there was a travel history to Pakistan 3 months before)

Based on World Health Organization recommendations, in terms of facing challenges caused by the COVID-19 pandemic, such as the disruption of the malaria rapid test kits supply, the shortage of health workers and personal protective equipment, as well as the limited facilities of the Intensive Care Unit (ICU), the diagnosis of malaria must always be considered in all cases of fever in malaria-endemic countries [21]. Considering the differential diagnosis of malaria infection other than SARS-CoV-2 infection can reduce the risk of morbidity and mortality due to delayed and inappropriate treatment.

Various clinical trials to determine the appropriate COVID-19 therapeutic regimen are still ongoing. The massive use of hydroxychloroquine to treat COVID-19 in Malaria endemic areas will further increase the risk of anti-malarial drug resistance [17]. In this case report, the patient was treated with Favipiravir, a nucleoside analogue antiviral efficacious in various clinical trials for treating mild-to-moderate COVID-19 [28]. In a 2021 review study, Zenchenko et al. concluded that nucleoside analogue antivirals could treat a parasitic infection, such as malaria caused by *P. falciparum*. Meanwhile, studies on the nucleoside analogue antiparasitic effect in vivax malaria are limited [29]. Artemisinin is a very potent anti-malarial drug and can overcome the problem of resistance to quinolones. Artemisinin is also able to inhibit endocytosis more strongly than chloroquine. Artesunate, a semisynthetic derivative of artemisinin, has lately attracted much attention to be tested as a COVID-19 therapy because of its anti-viral and anti-inflammatory effects through inhibition of Nuclear Factor kappa B (NF-kB) downregulation and protein synthesis in the early stages of viral replication [30]. Further research is required to study the role of artemisinin in treating COVID-19 and the prognosis of COVID-19 co-infection with malaria.

## Conclusion

This case report shows that considering the possibility of a secondary infection diagnosis other than COVID-19 in a patient who presents with fever can prevent delayed treatment that can worsen the disease outcome. Paying more attention to a history of travel to malaria-endemic areas, a history of previous malaria infection, and exploring anamnesis regarding the fever patterns in patients are important points in making a differential diagnosis of malaria infection during the COVID-19 pandemic. Hypercoagulopathy is another phenomenon that needs further analysis in Malaria and COVID-19 co-infection. Further research is required to study the role of artemisinin in treating COVID-19 and the prognosis of COVID-19 co-infection with malaria.

## Data Availability

Not applicable.

## Abbreviations

ACE-2: Angiotensin-converting enzyme-2
ALT: Alanine transferase
API: Annual parasite incidence
AST: Aspartate transaminase
COVID-19: Coronavirus disease 2019
CRP: C-reactive protein
DHP: Dihydroartemisinin-piperaquine
DIC: Disseminated intravascular coagulation
ICU: Intensive care unit
IL-6: Interleukin-6
IU: International unit
LDH: Lactate dehydrogenase
NETs: Neutrophil extracellular traps
NF-kB: Nuclear factor kappa B
RDT: Rapid diagnostic test
RT-PCR: Real-time polymerase chain reaction
SARS CoV-2: Severe acute respiratory syndrome coronavirus-2
TNF: Tumour necrosis factor
